# Shifting in the shadows: Morphofunctional variations of *Miconia sellowiana* Naudin (Melastomataceae) associated with cave environments

**DOI:** 10.1111/plb.70139

**Published:** 2025-11-11

**Authors:** G. H. Rosa, R. C. Cardoso, R. L. Ferreira, M. Souza‐Silva

**Affiliations:** ^1^ Programa de Pós‐graduação em Ecologia Aplicada Universidade Federal de Lavras, Instituto de Ciências Naturais Lavras Brazil; ^2^ Departamento de Ecologia e Conservação Universidade Federal de Lavras, Instituto de Ciências Naturais Lavras Brazil

**Keywords:** cave environment, functional traits, leaf anatomy, morphofunctional characteristics, morphological variation

## Abstract

- Caves present unique ecological conditions that influence the distribution and adaptation of species, yet studies on cave‐associated vegetation remain limited. This study investigated how cave conditions affect the functional traits of *Miconia sellowiana* Naudin (Melastomataceae), comparing individuals from the cave interior with those from the adjacent understory. Our objective was to understand how these environments influence the species' morpho‐functional characteristics and ecological relevance, aiming to identify physiological responses to the constraints of each habitat. Based on this, we hypothesize that caves act as distinct environmental filters compared to the understory, selecting for unique morphological and physiological variations.
- Leaf morpho‐functional traits were evaluated, including macroscopic dimensions (length, width, and leaf area) and microscopic characteristics, such as the anatomy of the central vein, mesophyll, and epidermis. Samples were fixed, processed for histological sections, and analysed by optical and electron microscopy. Statistical analysis included PCA to identify morpho‐functional patterns and Student's *t*‐tests/Wilcoxon tests to compare variables between habitats.
- Cave individuals had thinner leaves, with fewer layers of photosynthetic parenchyma, smaller relative phloem area in the central vein, lower stomatal density, and reduced leaf area and length compared to understory individuals.
- Low light availability, high humidity, shallow soils, and nutrient scarcity in caves likely limit the development of thicker leaves and affect stomatal density, vascular tissue, and leaf size. These results suggest that cave environments drive morpho‐functional and physiological variations in surrounding plants. This study fills gaps in the literature and highlights ecological mechanisms that sustain life in subterranean ecosystems.

## Graphical abstract

Cave environments filter plant traits, selecting thinner, smaller leaves with reduced photosynthetic and vascular structures.

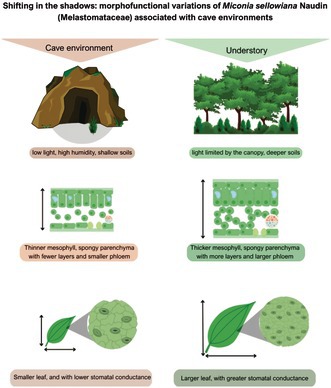

## INTRODUCTION

Phenotypic variation in plants in response to environmental conditions is essential for their ecological success, dispersal, and persistence (Khatri *et al*. 2023; Fartyal *et al*. 2025; Negi *et al*. 2025). Such variation can manifest through changes in morphological and physiological functional traits (Liu *et al*. 2020). Among these, leaves are particularly responsive, undergoing structural and functional modifications that reflect habitat‐specific constraints and opportunities (Khan *et al*. 2020; Khatri *et al*. 2022). Although genetically determined, these traits are strongly influenced by environmental conditions (Terashima 1992), such as water and nutrient availability, light intensity, and temperature (Wu *et al*. 2022), and may induce notable morpho‐anatomical variation, being particularly evident in plants capable of colonizing contrasting light microenvironments (Khatri *et al*. 2022; Negi *et al*. 2025).

Cave entrance environments represent a unique ecological context, with microclimates characterized by low light, high humidity, and altered nutrient dynamics. These environmental conditions can induce specialized trait expressions, potentially affecting leaf anatomy and physiological processes. Although caves share low light conditions with understory habitats, they present a unique combination of factors, high humidity, shallow soils, and nutrient scarcity, that make them ecologically distinct (Bai *et al*. 2020), and plant responses within these environments remain insufficiently studied (Monro *et al*. 2018). While some research indicates that plants adapt to low‐light conditions through strategies such as internode elongation and increased chlorophyll content to enhance light capture (Vandenbussche *et al*. 2005), these responses are often categorized as shade‐avoidance syndromes typical of heliophilous species. However, given that these are distinct environments, understory plants may respond to light limitation differently from plants in cave environments, as the latter are exposed to a more specific and constrained set of environmental factors, which can lead to unique adaptive responses beyond the typical strategies observed in the understory (Tao *et al*. 2015).

The vegetation surrounding cave entrances plays a vital role in regulating microclimate conditions and sustaining the cave ecosystem (Prous *et al*. 2015). By maintaining nutrient flow and providing habitat for endemic species, external vegetation contributes to the stability of the subterranean environment (Schneider *et al*. 2011). Deforestation and anthropogenic pressure in areas near caves negatively affect the associated fauna and may eliminate plant species adapted to these environments, many of which are potentially endemic, as reported in previous studies (Monro *et al*. 2018; Cardoso *et al*. 2022).

Studies on vegetation associated with cave entrance environments remain limited, particularly in tropical regions (Monro *et al*. 2018). This study uses *Miconia sellowiana* (Melastomataceae) as a model species to examine its morphological and anatomical traits in relation to physiological adaptations. Our objective was to understand how cave environments influence its functional traits by comparing individuals from cave interiors with those from adjacent understory habitats and identifying potential physiological responses to the specific conditions of each environment. We hypothesized that the cave environment acts as a selective filter that may favour expression of distinct structural traits. We expect to observe significant anatomical differences between the two environments. In the understory, we anticipate longer leaves with larger photosynthetic surface area and thickness, as well as higher stomatal density, related to the increased availability of light and nutrients. Additionally, we predict increased development of conductive tissues (xylem and phloem) in the understory, reflecting a higher abundance of conductive vessels in larger leaves. Lastly, we expect that protective tissues will be less developed in the caves, possibly associated with the typically lower herbivory pressure found in these environments.

## METHODS

### Study area

**Fig. 1:**
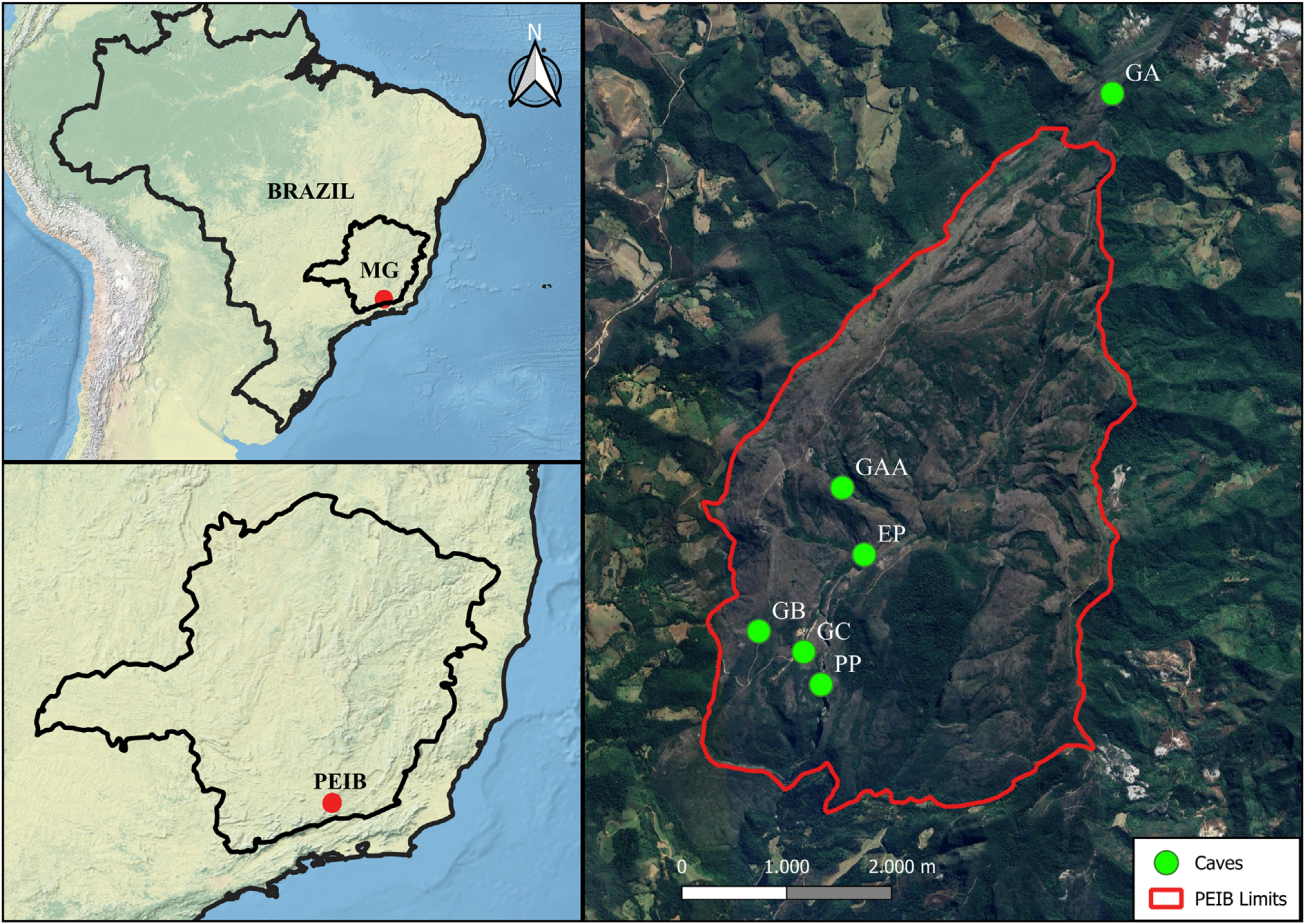
Location of the study area in Ibitipoca State Park (PEIB), Minas Gerais, Brazil. Green dots indicate sampled sites, represented by names of the respective caves, as well as the surrounding understory. The park boundary is highlighted in red. The top right and bottom right insets show the location of PEIB in Brazil and in Minas Gerais, respectively.

Ibitipoca State Park (PEIB) (Fig. 1) is a Strict Protection Conservation Unit (UCPI) located in Lima Duarte, Minas Gerais, Brazil. Despite its relatively small size (1,488 ha), the park hosts a remarkable concentration of caves (around 65), all developed in quartzite formations. Notably, the area includes some of the largest quartzite caves in Brazil (Rubbioli *et al*. 2019; Oliveira *et al*. 2024).

Although situated within the Atlantic Forest biome, the park's geological and geomorphological features sustain a wide variety of vegetation types (Oliveira‐Filho *et al*. 2013), including ombrophilous and cloud forests, high‐altitude shrublands, arboreal‐shrub and shrub savannas, as well as wooded and open grasslands. This mosaic of vegetation, together with the widespread presence of caves, dolines, and skylights, supports a rich and diverse plant community, with species capable of colonizing contrasting habitats, as observed for *M. sellowiana*.

### Field procedures

The species *M. sellowiana* is commonly found in the understory of the park's forested environments as well as at cave entrances. To compare morphological variations in its leaves, a total of 21 individuals were sampled from both habitats. Ten individuals were collected from caves (GAA = 3, GA = 1, GB = 2, GC = 1, EP = 1, PP = 2) and 11 from the understory (GAA = 3, GA = 2, GB = 1, GC = 1, EP = 2, PP = 2; Table 1). In this study, we treated understory and cave as two distinct environments, without considering individual caves as separate sampling units. Therefore, variation in the number of individuals collected per cave did not affect our interpretation, since comparisons were made at the habitat level. The number of individuals sampled in each cave reflected the local availability of *M. sellowiana*, and all accessible individuals were included to maximize sample size. Although the understory contained only one more individual than the cave environment, we opted to retain it given the overall limited availability of plants.

**Table 1:** Location of sampled individuals in the understory and internal cave regions.

cave	understory (m)	internal (m)	latitude	longitude	altitude (m a.s.l.)
GAA	5, 25, 30	20, 20, 25	21°41′48.05″S	43°53′33.79″W	1552
GA	2	20, 50	21°39′32.07″S	43°51′20.67″W	1554
GB	15, 15	7	21°42′33.10″S	43°53′48.48″W	1444
GC	1	1	21°42′28.99″S	43°53′41.21″W	1355
EP	1	20, 40	21°42′12.31″S	43°53′26.37″W	1391
PP	20, 30	50, 50	21°42′52.56″S	43°53′46.63″W	1268

The distances indicate the position of each individual relative to the cave entrance, and the number of distances represents the number of individuals collected. (GAA = Gruta Arco do Arlete; GA = Gruta das Andorinhas; GB = Gruta das Bromélias; GC = Gruta dos Coelhos; EP = Espelhos; PP = Ponte de Pedra). (Reference datum: SIRGAS 2000).

Collections were carried out in selected caves based on speleotopographic maps, which indicated the presence of vegetation at their entrances. Sampling sites were chosen based on the occurrence of *M. sellowiana* in both hypogean (cave) and epigean (understory) zones adjacent to cave entrances. For each sampled individual, the distance from the cave entrance and geographic coordinates were recorded (Table 1).

Leaf collection followed a standardized protocol, selecting leaves from the third node in an apex‐to‐base direction. A total of 10 leaves per individual were collected from both environments, resulting in 210 leaves for analysis. Additionally, some whole individuals were collected for taxonomic identification. For anatomical and leaf area analyses, five leaves per individual were fixed and preserved in 70% ethanol, totaling 105 leaves. The remaining leaves were used for morphometric measurements in the field.

### Morphological analysis and anatomy

For the analysis of macroscopic functional morphometric traits, five leaves per individual were measured fresh in the field using a calliper to determine leaf length and width. Subsequently, in the laboratory, three leaves per individual were removed from ethanol, dried, and scanned to calculate leaf area using a Samsung SCX 4600 flatbed scanner at 300 dpi with a centimetre scale. Leaf area and other measurements were obtained using ImageJ software, calibrated based on the centimetre scale; pixels were assumed square (pixel aspect ratio = 1.0), and no further adjustments to resolution were applied.

After scanning, the leaves were re‐stored in 70% ethanol for the preparation of temporary slides. Sections of the plant material were manually cross‐sectioned at the mid‐region of the leaf, cleared with 100% sodium hypochlorite, rinsed with distilled water, and stained with Safrablau (1% safranin and 0.1% Astra blue), which differentiates primary cell walls (blue) from secondary cell walls (red) (Kraus & Arduin 1997). The samples were then mounted in 50% glycerinated water (Johansen 1940). At least three slides per individual were prepared, each containing cross‐sections from five leaves.

In the cross‐sections of the mid‐region of the leaf, measurements were taken for area, diameter, and perimeter of various tissues based on three slides per individual, including the central vein, total xylem, phloem, collenchyma, and individual xylem vessels. Additionally, for the central vein and individual xylem vessels, circularity values were measured, providing insights into water transport efficiency and plant adaptation to environmental conditions (Andrade *et al*. 2024). Cross‐sections were also used to assess total mesophyll thickness, adaxial epidermis thickness, external wall thickness of the adaxial epidermis, total parenchyma thickness in the mesophyll, number of palisade parenchyma layers, and number of spongy parenchyma layers (Andrade *et al*. 2024).

To determine the density of epidermal cells (adaxial and abaxial surfaces) and stomatal density on the abaxial surface (*M. sellowiana* being a hypostomatic species), temporary slides were prepared using paradermal sections. Rectangular sections were taken from the central‐marginal region of the leaves, with each leaf sectioned laterally and placed in 2 mL microtubes containing 100% sodium hypochlorite. The samples were then incubated in an oven at 60°C until fully cleared. The epidermal layers, which separated from the mesophyll during this process (Bersier & Bocquet 1960), were rinsed with distilled water, stained with 1% safranin, and mounted in 50% glycerinated water (Johansen 1940).

For each longitudinal section, the number of epidermal cells on both the adaxial and abaxial surfaces was recorded. Additionally, perimeter, area, diameter, and average stomatal circularity were measured based on a sample of three stomata per section, and each individual was analysed in three separate repetitions, resulting in a minimum of nine stomata measured per individual. This approach captured the natural variation among leaves, which is reflected in the low observed standard deviation in our measurements. Stomatal density was expressed as the number of stomata per unit area at 20× objective magnification. Histological slides were photographed using an optical microscope equipped with a ZEISS Axiocam 105 colour digital camera.

Furthermore, scanning electron microscopy (SEM) images were obtained using a Hitachi TM4000 tabletop SEM. Cross‐sections of the leaves were analysed to examine mesophyll and central vein structures, while small sections of the leaf surface were used to observe the abaxial epidermis and stomata.

### Definition of morphofunctional groups

To effectively organize and interpret the measured parameters, they were grouped into morphofunctional categories based on their physiological and structural roles in leaves. This enabled a more comprehensive evaluation of each parameter's relevance to key processes such as photosynthesis, water and nutrient transport, protection against environmental factors, and mechanical support, as previously proposed to describe the functional significance of morphofunctional traits (Vale *et al*. 2025).

Based on the above criteria, the ‘Photosynthesis’ group included total mesophyll thickness (TMT), total parenchyma thickness (TMPT), number of palisade parenchyma layers (NPPL), number of spongy parenchyma layers (NSPL), and the proportion of parenchyma thickness relative to total mesophyll thickness (PTMT). Stomatal traits included stomatal area (SA), stomatal diameter (SD), stomatal circularity (SC), and stomatal density (SQ). Additionally, macroscopic leaf measurements, such as leaf width (LW), leaf length (LL), and leaf area (LA) were collected.

The ‘Conduction’ group included central vein area (ACV), total xylem area (TAXCV), phloem area (APCV), xylem vessel area (AXV), and xylem vessel circularity (CXV). Additionally, we considered the proportion of phloem and xylem areas relative to the central vein (PTPACV and PXVACV). The ‘Protection’ group comprised epidermal‐related measurements, such as outer mesophyll wall thickness (OWT), mesophyll epidermal thickness (ET), abaxial epidermal quantity (AEQ), and adaxial epidermal quantity (ADQ). The ‘Support’ group included collenchyma area in the central vein (CAV) and the proportions of xylem and collenchyma areas (PXCA and PCCA).

### Statistical analysis

To investigate variations in morphofunctional traits between understory and cave environments, we performed Principal Components Analysis (PCA). This approach reduced data dimensionality, condensing measured variables into principal axes, thereby facilitating the visualization and interpretation of differences and similarities among groups. Ellipses with a 95% confidence interval around the centroid were used to represent data dispersion and identify potential clustering patterns.

Initially, we conducted a comprehensive PCA that included all measured parameters to obtain a preliminary assessment of the separation between individuals from cave and understory environments. The analyses were conducted using the prcomp function, with pre‐scaled variables, implemented in the R package ‘factorextra’. The significance of the principal axes and variables was evaluated using the ‘PCAtest’ package, which assesses the global statistical relevance of the PCA, the significance of each axis, and the individual contribution of variables through permutation tests. Biplot graphs and confidence ellipses were generated using the ‘ggplot2’ package (Wickham 2011).

Following the PCA, we applied Student's *t*‐test to identify significant differences in mean variable values between cave and understory environments. The *t*‐test was selected as it is appropriate for comparing means between two independent samples, corresponding to the two studied habitats. Before applying the *t*‐test, we verified normality and homogeneity of variances using the Shapiro–Wilk and Levene tests, respectively. The number of palisade parenchyma layers parameter was excluded from the analyses because no variation in the number of layers was observed in any individual, except one. Variables that did not meet these assumptions (area of central vein, area of phloem in central vein, area of xylem vessel, and circularity of xylem vessel in the ‘Conduction’ group; abaxial epidermis quantity in the ‘Protection’ group; Collenchyma area of the central vein in the ‘Support’ group) were log‐transformed. When, even after transformation, the assumptions for the parametric test were not satisfied, we used the non‐parametric Wilcoxon test (required for the area of xylem vessel, circularity of xylem vessel, and adaxial epidermis quantity parameters) to ensure reliability of the results. All statistical analyses were performed in R software (Core Team 2024) using the ‘*t*.test’ function, while mean comparison graphs were generated using the ‘ggplot2’ package (Wickham 2011).

## RESULTS

### Group separation based on PCA

**Fig. 2:**
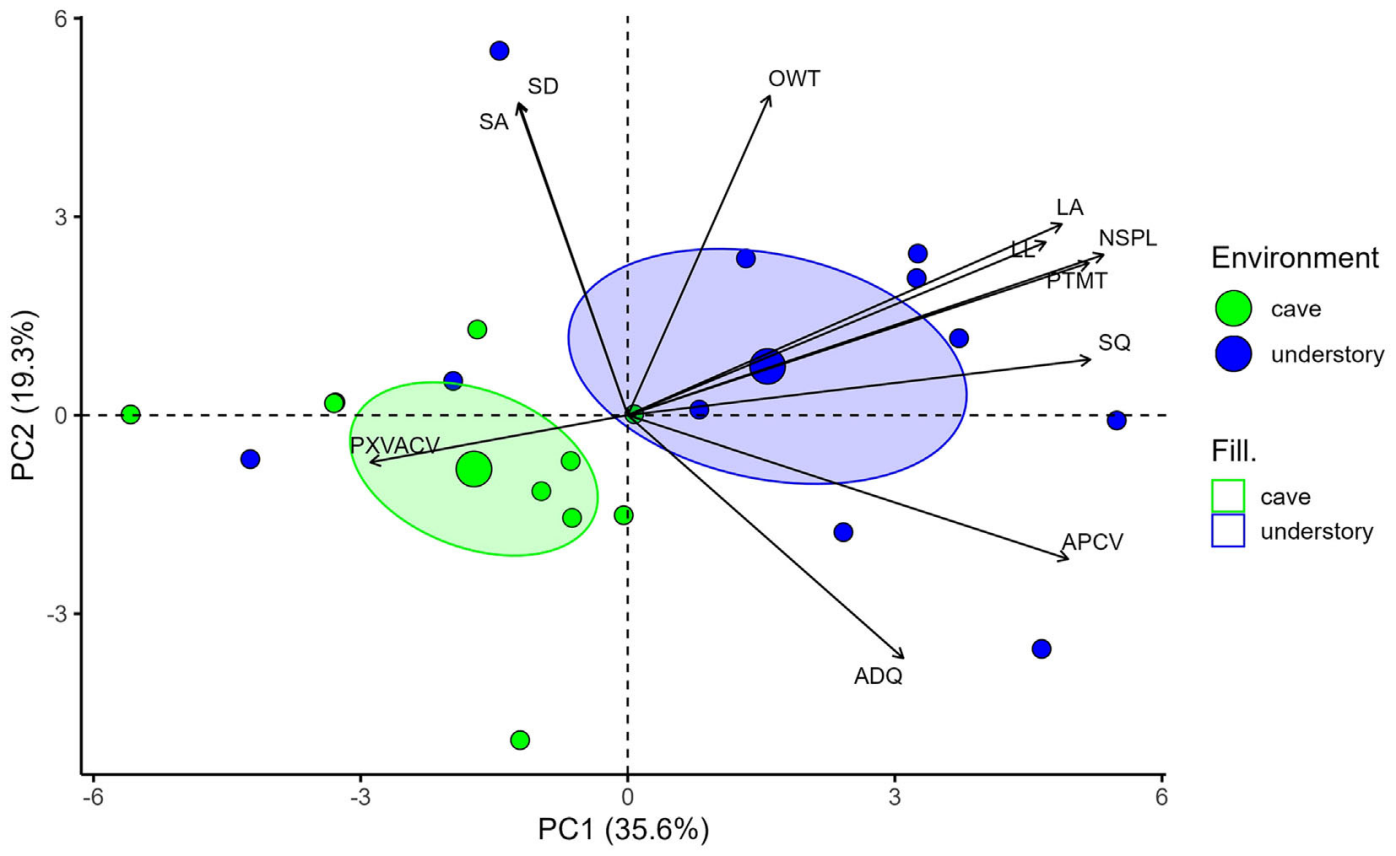
PCA of leaf morpho‐functional traits in *M. sellowiana* individuals from understory (blue) and cave (green) environments. Each point represents one individual, with point size proportional to the individual's contribution to trait variation. Ellipses represent 95% confidence intervals for each group. Arrows indicate traits with correlation coefficients |*r*| > 0.5 with either PC1 or PC2. The direction and length of each arrow indicate the sign and strength of the correlation. Traits include: Number of spongy parenchyma layers (NSPL), Proportion of total parenchyma thickness to total mesophyll (PTMT), Stomata quantity (SQ), Area of phloem in central vein (APCV), Leaf area (LA), Leaf length (LL), Mesophyll outer wall thickness (OWT), Stomatal area (SA), Stomatal diameter (SD), Adaxial epidermis quantity (ADQ), Proportion of xylem vessel area to central vein (PXVACV) together; pc1 and pc2 explain 54.9% of the total variation in traits.

Principal Components Analysis (PCA) of the complete dataset identified two main axes that together explained 54.9% of the total variance in leaf traits (Fig. 2). The first axis (PC1, 35.6%) primarily represented structural investment in leaves, exhibiting significant positive loadings (correlations >0.75) for total mesophyll thickness (TMT), number of spongy parenchyma layers (NSPL), leaf area (LA), and proportion of phloem to central vein area (PTPACV), with moderate contributions from leaf length (LL) and area of phloem in the central vein (APCV) (Table 2). The second axis (PC2, 19.3%) captured variation in epidermal and protective attributes, showing strong positive loadings for outer wall thickness (OWT) and stomatal area (SA), and negative loadings for adaxial epidermis quantity (ADQ) and area of xylem vessel (AXV), highlighting the contrasting distribution of traits related to leaf surface structure versus internal vascular architecture (Table 2). Two understory individuals clustered closer to the cave group in the PCA ordination (Fig. 2). Despite the overall separation between habitats, this pattern can be explained by the fact that these samples were collected in understory areas adjacent to Ponte Pedra and Espelhos caves, both located near bodies of water. This proximity may have created microenvironments with high humidity, similar to conditions typically found at cave entrances. Consequently, these individuals exhibit leaf trait patterns closer to those of cave plants than to those from other understory sites.

**Table 2:** Correlations between leaf morphofunctional traits of *M. sellowiana* and the first two significant axes (PC1 and PC2) of the PCA.

traits	abbreviation	correlation		*P*	
		PC1	PC2	PC1	PC2
Total mesophyll thickness	TMT	0.48	0.67	0.029	0.001
Total mesophyll parenchyma thickness	TMPT	0.62	0.62	0.003	0.003
Proportion of parenchyma thickness to mesophyll	PTMT	0.80	0.36	0.000	0.114
Number of spongy parenchyma layers	NSPL	0.83	0.38	0.000	0.094
Leaf length	LL	0.73	0.40	0.000	0.069
Leaf area	LA	0.75	0.45	0.000	0.043
Mesophyll outer wall thickness	OWT	0.25	0.75	0.282	0.000
Mesophyll epidermis thickness	ET	−0.504	0.25	0.020	0.268
Abaxial epidermis quantity	AEQ	0.73	−0.427	0.000	0.054
Adaxial epidermis quantity	ADQ	0.48	−0.567	0.028	0.007
Central vein area	ACV	0.65	−0.478	0.001	0.028
Total area of xylem in central vein	TAXCV	0.86	−0.362	0.000	0.107
Area of phloem in central vein	APCV	0.76	−0.335	0.000	0.137
Area of xylem vessel	AXV	0.23	−0.517	0.308	0.017
Circularity of xylem vessel	CXV	−0.090	0.36	0.698	0.114
Proportion of phloem to central vein area	PTPACV	0.71	−0.045	0.000	0.845
Proportion of xylem vessel area to central vein	PXVACV	−0.446	−0.110	0.043	0.636
Collenchyma area of the central vein	CAV	0.72	−0.474	0.000	0.030
Proportion of total xylem to central vein	PXCA	0.71	0.03	0.000	0.888
Proportion of collenchyma area to central vein	PCCA	0.40	−0.040	0.074	0.862
Leaf width	LW	0.59	0.20	0.005	0.384
Stomatal quantity	SQ	0.80	0.13	0.000	0.574
Stomatal area	SA	−0.190	0.73	0.409	0.000
Stomatal diameter	SD	−0.189	0.73	0.412	0.000
Stomatal circularity	SC	0.19	0.01	0.418	0.972

The ‘Measurement Description’ column describes the anatomical or morphological trait assessed; ‘Variable’ indicates the corresponding abbreviation used in the analysis; ‘Correlation’ presents the correlation coefficients of each variable with PC1 and PC2; and ‘*P*‐value’ is the statistical significance of these correlations. Higher absolute correlation values (positive or negative) reflect a larger contribution of the variable to the variation explained by each axis, and therefore, to the separation of groups along the respective principal component. Proportion of variance explained (PVE): PC1 = 35.6%, PC2 = 19.3%.

### Morphofunctional group analysis

**Fig. 3:**
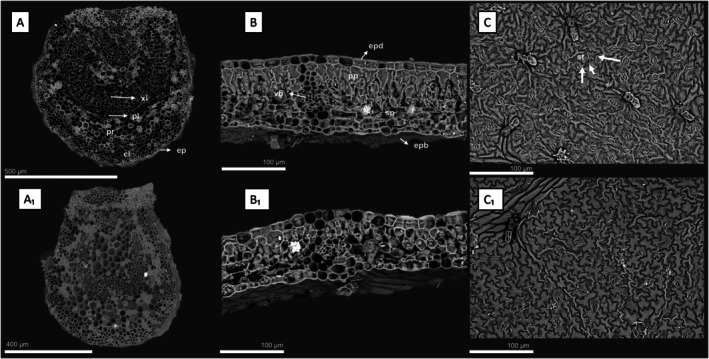
Scanning electron microscopy (SEM) histological sections highlighting the leaf regions analysed for anatomical parameter measurements. cl, collenchyma; ep, epidermis; epb, abaxial epidermis; epd, adaxial epidermis; pl, phloem; pp, palisade parenchyma; pr, filling parenchyma; sp, spongy parenchyma; st, stomata; vb, vascular bundle; xl, xylem. (A) Central leaf vein of an individual from the epigean (understory) region; (A_1_) Central vein of an individual from the hypogean (cave) region; (B) Leaf mesophyll of an individual from the epigean region; (B_1_) Leaf mesophyll of an individual from the hypogean region; (C) Epidermis highlighting stomata in an individual from the epigean region; (C_1_) Epidermis highlighting stomata in an individual from the hypogean region.

**Fig. 4:**
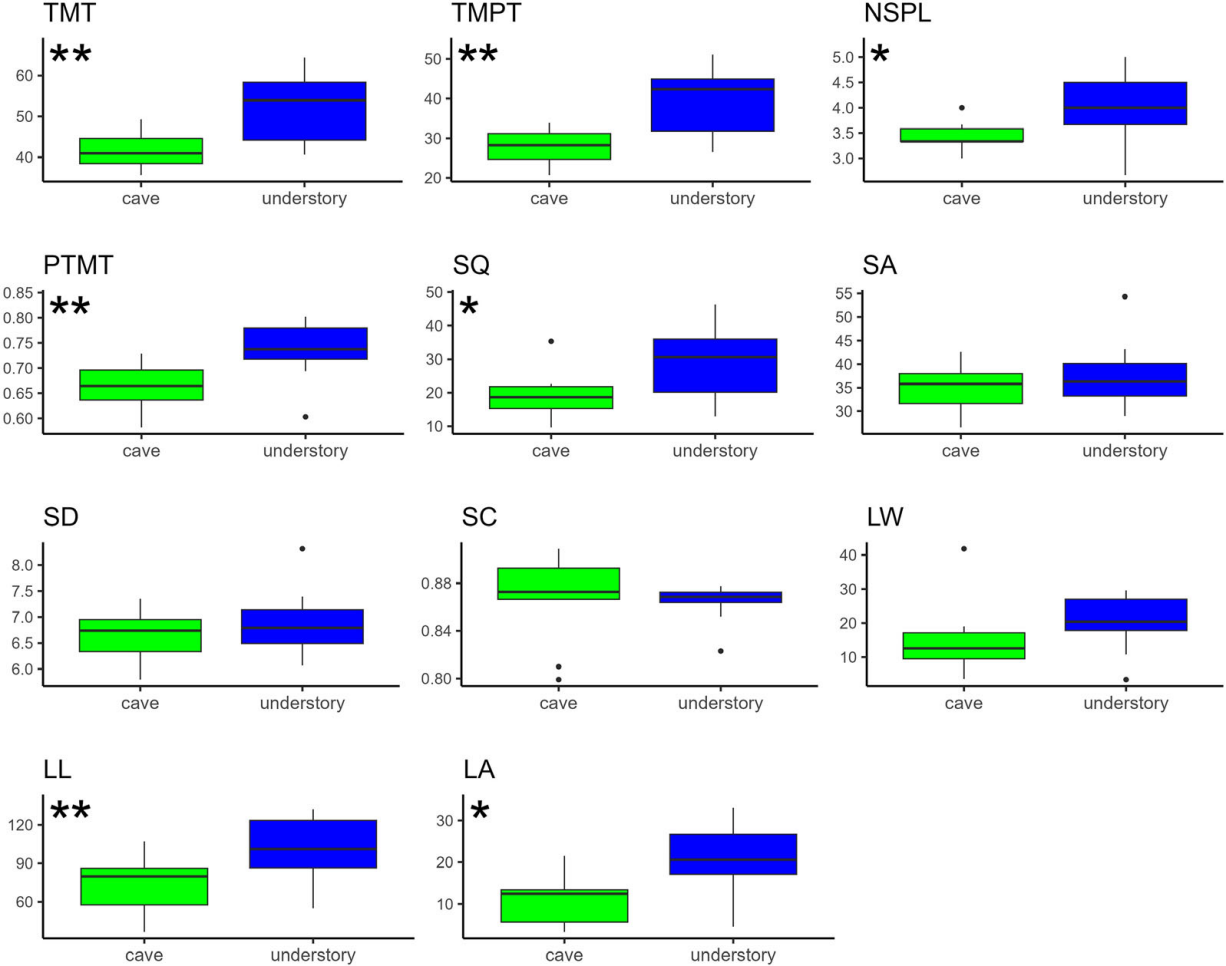
Boxplot highlighting differences between means of each parameter for the ‘Photosynthesis’ group between the understory (blue quantiles) and cave (green quantiles) environments. Boxplot marked with ‘*’ is significant difference with Student's *t*‐test (*P < 0,05, **P < 0,01). Total mesophyll thickness (TMT); total mesophyll parenchyma thickness (TMPT); number of spongy parenchyma layers (NSPL); proportion of total parenchyma thickness to total mesophyll (PTMT); stomatal density (SQ); stomatal area (SA); stomatal diameter (SD); stomatal circularity (SC); leaf width (LW); leaf length (LL); and leaf area (LA).

The *t*‐tests revealed several anatomical parameters with significantly higher mean values (*P* < 0.05) in sub‐canopy plants compared with cave plants, with these differences clearly illustrated in the SEM micrographs (Fig. 3). Within the ‘Photosynthesis’ group (Fig. 4), significant differences were detected for total mesophyll thickness (TMT), total mesophyll parenchyma thickness (TMPT), proportion of parenchyma to mesophyll thickness (PTMT), number of spongy parenchyma layers (NSPL), leaf length (LL), leaf area (LA), and stomatal density (SQ) (Fig. 3B,C, B_1_,C_1_). Other stomatal traits, including stomatal area (SA), stomatal diameter (SD), and stomatal circularity (SC), did not differ significantly between environments (Table 3).

**Table 3:** Mean values of leaf morpho‐functional traits of *M. sellowiana* individuals collected from cave and understory environments.

trait	abbreviation	physiological group	cave	UNDERSTORY	*P*‐value	test value	DF
Total mesophyll thickness (μm)	TMT	Photosynthesis	41.86	51.45	0.004	−3.36	15.99
Total mesophyll parenchyma thickness (μm)	TMPT	Photosynthesis	27.66	38.21	0.002	−3.74	15.22
Number of spongy parenchyma layers	NSPL	Photosynthesis	3.40	4.03	0.016	−2.74	14.07
Number of palisade parenchyma layers	NPPL	Photosynthesis	1.00	1.05	‐	‐	‐
Proportion of total parenchyma thickness to total mesophyll	PTMT	Photosynthesis	0.65	0.73	0.003	−3.39	18.65
Leaf width (cm)	LW	Photosynthesis	14.64	20.40	0.188	−1.37	16.69
Leaf length (cm)	LL	Photosynthesis	72.88	101.94	0.010	−2.82	18.92
Leaf area (cm^2^)	LA	Photosynthesis	13.67	20.39	0.010	−2.9	17.52
Stomatal area (μm)	SA	Photosynthesis	35.23	37.70	0.356	−0.95	17.80
Stomatal diameter (μm)	SD	Photosynthesis	6.67	6.90	0.364	−0.93	18.38
Stomatal circularity (μm)	SC	Photosynthesis	0.86	0.86	0.759	0.31	11.83
Stomatal quantity	SQ	Photosynthesis	19.43	29.48	0.019	−2.59	17.23
Area of central vein (mm^2^)	ACV	Conduction	93.76	107.28	0.439	−0.79	18.93
Total area of xylem in central vein (mm^2^)	TAXCV	Conduction	5.07	6.81	0.111	−1.67	18.31
Area of phloem in central vein (mm^2^)	APCV	Conduction	9.02	12.91	0.127	−1.62	14.65
Area of xylem vessel (μm^2^)	AXV	Conduction	42.44	39.33	0.512	45 (w)	‐
Circularity of xylem vessel	CXV	Conduction	0.90	0.90	0.251	38 (w)	‐
Proportion of total phloem area to central vein	PTPACV	Conduction	0.09	0.11	0.021	−2.54	16.65
Proportion of xylem vessel area to central vein	PXVACV	Conduction	< 0.001	< 0.001	0.147	1.52	18.33
Mesophyll outer wall thickness (μm)	OWT	Protection	1.67	1.93	0.085	−1.82	18.48
Mesophyll epidermis quantity (μm)	ET	Protection	6.61	6.83	0.714	−0.37	18.96
Abaxial epidermis quantity	AEQ	Protection	157.29	183.93	0.196	−1.34	18.55
Adaxial epidermis quantity	ADQ	Protection	154.46	176.39	0.557	46 (w)	‐
Collenchyma area of the central vein (μm^2^)	CAV	Support	9.66	9.85	0.24	−1.21	18.18
Proportion of total xylem area to central vein area	PXCA	Support	0.05	0.06	0.07	−1.94	15.98
Proportion of total collenchyma area to central vein area	PCCA	Support	0.17	0.18	0.17	−1.41	18.68

The evaluated traits include anatomical and morphological leaf characteristics. Each trait was classified into a physiological group according to its primary function: Photosynthesis, Conduction, Protection, or Support. The ‘Cave’ and ‘Understory’ columns present the mean values observed in each environment. The ‘*P*‐value’ column indicates statistical significance, with *P* < 0,05 representing statistically significant differences between environments. The ‘Test Value’ column indicates results from the Student's *t*‐test, while values marked with ‘(w)’ correspond to parameters analysed using the Wilcoxon test. The ‘DF’ column reports degrees of freedom for the *t*‐test. Rows with ‘‐’ indicate parameters without measurements.

**Fig. 5:**
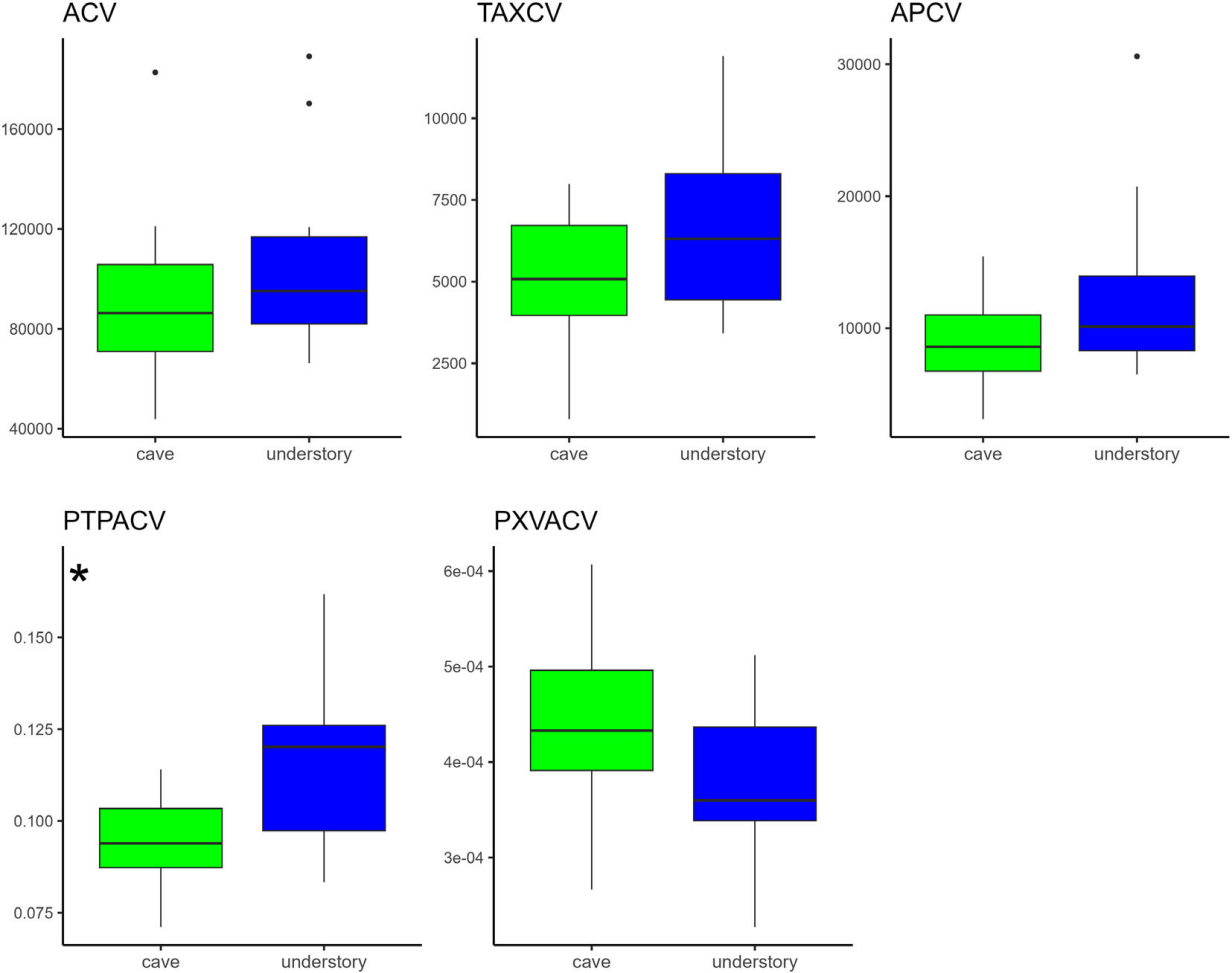
Boxplot highlighting differences between the means of each parameter for the ‘Conduction’ group between the understory (blue quantiles) and cave (green quantiles) environments. Boxplot marked with ‘*’ show significant differences for Student's *t*‐test (*P < 0,05, **P < 0,01). Area of central vein (ACV); total area of xylem in central vein (TAXCV); area of phloem in central vein (APCV), area of xylem vessel (AXV); circularity of xylem vessel (CXV); proportion of total phloem area to central vein (PTPACV); proportion of xylem vessel area to central vein (PXVACV).

In the ‘Conduction’ group, *t*‐tests indicated generally higher mean values in sub‐canopy plants than in cave plants (Fig. 5), although only the proportion of phloem to central vein area (PTPACV) showed a statistically significant difference (Fig. 3A,A1). Other conduction‐related traits, including central vein area (ACV), total xylem area (TAXCV), proportion of xylem to central vein area (PXVACV), and phloem area (APCV), were higher in sub‐canopy leaves but not significantly different (Table 3). Xylem vessel area (AXV) and circularity (CXV), analysed using non‐parametric Wilcoxon tests due to non‐normal distributions, also exhibited no significant differences (Table 3). Within the ‘Protection’ and ‘Support’ groups, both *t*‐tests and Wilcoxon tests revealed consistently higher mean values in sub‐canopy plants relative to cave plants across all measured traits; however, none of these differences were statistically significant (Table 3).

## DISCUSSION

Our results indicate that cave habitats are associated with pronounced morphofunctional variations in leaf traits. Although cave entrances share low light availability with other shaded environments, such as tropical forest understories, deep canyons, and rocky slopes beneath dense canopy, they also present a unique combination of intrinsic characteristics. These include greater microclimatic stability (Cardoso *et al*. 2022), reduced nutrient availability (Souza‐Silva *et al*. 2021), and consistently high humidity levels (Mammola *et al*. 2016). The studied model species inhabiting cave environments exhibited distinctive leaf features, including thinner mesophyll layers, fewer spongy parenchyma strata, smaller phloem vessel areas, lower stomatal densities, and shorter, narrower leaves. Key abiotic factors, such as humidity, nutrient availability (Witkowski & Lamont 1991), and light intensity (Nobel *et al*. 1975; Upadhyaya & Furness 1994), act as strong environmental filters influencing plant trait expression in these habitats. These constraints directly affect attributes such as leaf area, thickness (Turner 1994), and photosynthesis rate (Romero‐Aranda *et al*. 1997), thereby drive the observed differentiation in leaf traits between cave and non‐cave environments. Our study contributes novel evidence that cave environments function as environmental filters influencing the anatomical and functional traits of plants.

The differences in the anatomical and morphological leaf traits of *M. sellowiana* between cave entrance (hypogean) and understory (epigean) environments highlight the contrasting environmental filters that shape leaf phenotypes. This divergence was particularly evident in the pronounced separation of the ellipses representing each environment, underscoring the contrasting environmental filters shaping leaf phenotypes (Stotz *et al*. 2021). The first axis (PC1), strongly correlated with traits such as leaf size, mesophyll thickness, and tissue density, primarily distinguished individuals exhibiting higher investment in structural support and photosynthetic capacity, features characteristic of understory plants. In contrast, the second axis (PC2), associated with epidermal thickness and stomatal traits, and some conduction and support attributes, represents a functional trade‐off between surface protection and investment in vascular conduction and structural support, indicating that plants may prioritize either protective structures and larger stomata or conductive and support tissues. These findings support the notion that environmental conditions exert a strong influence on the expression of functional traits, shaping plant phenotypes in response to specific habitat constraints (Gratani *et al*. 2006; Fartyal *et al*. 2025), including elevation (Choler 2005), water availability (Matos *et al*. 2021; Kramp *et al*. 2022), and soil properties (Buri *et al*. 2017).

Morphological and functional traits related to the ‘Photosynthesis’ group showed significant differences between cave and understory environments, mostly associated with PC1. In cave plants, the reduced mesophyll thickness may be associated with low light availability, which directly affects photosynthesis by limiting light capture and reducing the efficiency of ATP and NADPH production, both essential for the Calvin cycle and carbon fixation. Additionally, the lower number of spongy parenchyma layers in cave plants likely reflects adaptation to reduced light conditions. This tissue, with its intercellular spaces, facilitates CO_2_ diffusion within the leaf, a crucial process in environments with limited light (Vogelmann *et al*. 1996). However, in caves, the overall demand for CO_2_ diffusion is probably lower because of the inherent photosynthetic limitations. The number of palisade parenchyma layers did not vary between environments (except for one individual) and, as noted by Ivanova & P’yankov (2002), in shaded environments, spongy parenchyma can be as efficient as, or even more efficient than, palisade parenchyma in CO_2_ conductance.

Although diffuse light is the primary light source in both cave entrances and forest understories, these environments differ markedly in light filtering dynamics and nutrient availability. In forest understories, natural disturbances such as canopy openings can increase light penetration, thereby enhancing photosynthetic activity and promoting the development of larger, thicker leaves (Bazzaz 1983). In contrast, cave entrances offer a more stable microenvironment, largely insulated from external climate fluctuations and disturbances (Liang *et al*. 2019). Beyond light limitation, another major constraint in cave environments is their oligotrophic nature. The low nutrient availability, combined with shallow soils, may restricts the development of thicker leaves and affect plant metabolism and photosynthetic efficiency (De Paula *et al*. 2019). The combination of nutrient‐poor soils and limited light availability constraints may result in the thinner leaves observed in cave environments (Northup *et al*. 2001).

The analysis of traits within the ‘Conduction’ group revealed a statistically significant difference in the proportion of phloem area relative to the central vein. In understory plants, the phloem occupies approximately 3% more of the central vein area compared to the cave‐dwelling plants. This finding is consistent with the results from the ‘Photosynthesis’ group, which indicated larger total mesophyll thickness in understory plants. A thicker mesophyll reflects a higher density of photosynthetic cells, resulting in increased sugar production during photosynthesis. The corresponding rise in photosynthetic activity creates greater demand for the transport of assimilates, which occurs via the phloem (Huang *et al*. 2022). The smaller proportion of phloem in the central veins of cave plants aligns with their smaller leaf size and thinner parenchyma, suggesting that these plants likely have lower photosynthesis rates than those growing in external environments such as the understory.

The PC2 was associated with epidermal thickness and stomatal traits, corresponding to both the ‘Photosynthesis’ and ‘Protection’ groups. In addition to these relationships, significant differences were also observed in leaf area and length, as well as stomatal density. As previously discussed, the limited light availability in caves can lead to lower photosynthetic activity and consequently a reduced demand for CO_2_ uptake and gas exchange (Terashima 1992; Sarbu *et al*. 1996). Additionally, the consistently high relative humidity in caves may promote the formation of a boundary layer around leaf surfaces, further limiting gas exchange and reducing the necessity for high stomatal density. Another important factor is the nature of cave soils, which are typically shallow, nutrient‐poor, and possess low water retention capacity. These conditions may favour traits associated with water conservation, such as reduced stomatal density (Van Der Waal *et al*. 2009). Furthermore, certain traits from the Conduction and Support groups also correlate with PC2, indicating a functional trade‐off: plants can prioritize epidermal protection and larger stomata or, alternatively, invest in conduction and structural support tissues. This pattern reflects the specific environmental pressures of cave environments, where low light availability reduces the demand for intense gas exchange (Terashima 1992; Sarbu *et al*. 1996), while poor soil quality and high relative humidity favour strategies aimed at resource conservation and surface protection (Pentecost & Zhaohui 2001).

Although morphofunctional traits related to the ‘Protection’ and ‘Support’ groups exhibited slightly greater mean values in understory plants compared to cave‐dwelling individuals, these differences were not statistically significant. This likely reflects the predominantly shaded conditions of both environments, where leaf architectural variations are more pronounced in comparisons between sun‐exposed and shaded plants (Poorter & Werger 1999). Similarities in protective tissues may also be related to lower herbivory pressure in the understory, due to reduced young leaf density and limited nitrogen availability, which decrease leaf attractiveness to herbivores (Ribeiro & Basset 2007), balancing the pressure experienced by plants in cave habitats, which already have low faunal density (Cardoso *et al*. 2022). Consequently, this species maintains relatively thin leaves and prioritizes resource allocation to photosynthetic tissues over structural support or protective tissues. Moreover, in low‐light habitats, the continuous redistribution of resources throughout growth is essential to minimize self‐shading, although this strategy can be energetically costly (King 1991). Thus, despite differences in leaf size, structural requirements remain similar across the two environments.

## CONCLUSIONS

The results of this study underscore the complex ecological interactions that shape the functional, anatomical, and physiological adaptations of plants in cave environments. Flora inhabiting extreme ecosystems, such as caves, offer a unique opportunity to study the physiological mechanisms that enable survival under harsh conditions, including low light availability and nutrient scarcity (Petrik *et al*. 2022). When preserved, the vegetation surrounding caves ensures microclimate stability, moisture retention, erosion control, and the input of organic debris that sustains subterranean communities (Schneider *et al*. 2011). In addition, it supports cave‐dependent organisms such as bats, which provide key ecosystem services including pollination, seed dispersal, and agricultural pest control (Mammola *et al*. 2019). These interactions are vital for maintaining cave ecosystem functionality and enhancing its resilience against both climatic fluctuations and human‐induced disturbances. Cave plants can be used as model systems to predict how other species may respond to climate change scenarios involving extreme environmental stress (Axelsson *et al*. 2020). Their adaptive traits also reinforce their potential for restoration projects in degraded karst landscapes affected by human activities, such as mining, urbanization, livestock grazing, and monoculture, where shallow soils and harsh environmental conditions hinder natural regeneration (Zheng *et al*. 2024).

## Author Contributions

Conceptualization: GHR, RCC, MSS; Methodology: GHR, RCC; Validation: RCC, MSS, RLF; Formal Analysis: GHR, RCC; Resources: GHR, RCC, MSS, RLF; Writing—Original Draft: GHR, RCC; Writing—Review & Editing: MSS, RLF, RCC; Supervision: RLF, MSS; Project Administration: GHR, RCC; Funding Acquisition: GHR, RCC. All authors have read and agreed to the published version of the manuscript.

## Supporting information


**Figure S1.** Box‐plot highlighting the means of each parameter for the ‘Protection’ group between the understory (blue quantiles) and cave (green quantiles) environments. Mesophyll outer wall thickness (OWT); mesophyll epidermis quantity (ET); abaxial epidermis quantity (AEQ); adaxial epidermis quantity (ADQ).

**Figure S2.** Box‐plot highlighting the means of each parameter for the ‘Support’ group between the understory (blue quantiles) and cave (green quantiles) environments. Collenchyma area of the central vein (CAV); proportion of total xylem area to central vein area (PXCA); proportion of total collenchyma area to central vein area (PCCA).
