# Supplementary figures and images for: Shifting in the shadows: Morphofunctional variations of Miconia sellowiana Naudin (Melastomataceae) associated with cave environments
Source: Plant Biol (Stuttg). 2025 Nov 11;28(2):441–51. doi: 10.1111/plb.70139 (PMC12884021; doi:10.1111/plb.70139)

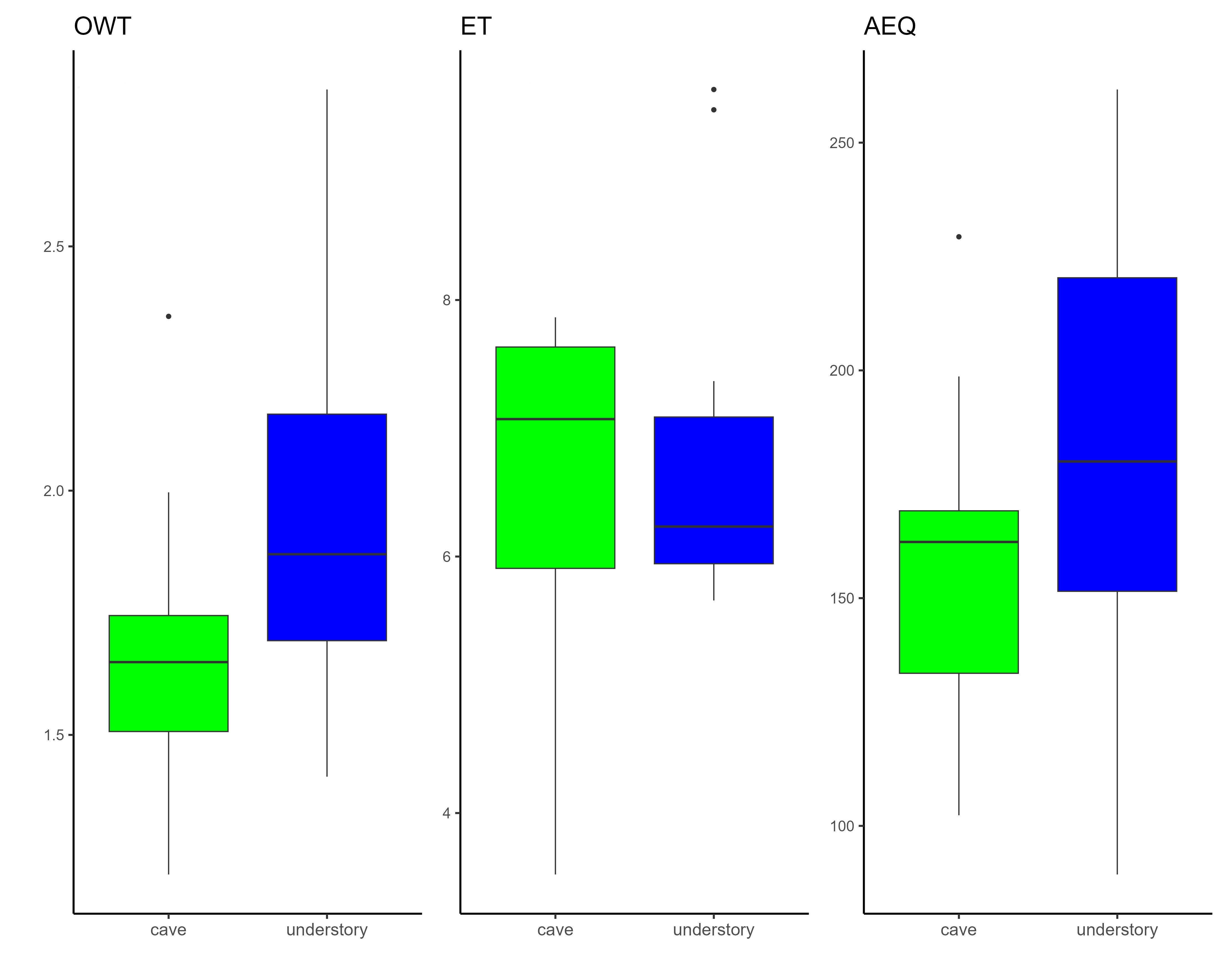

Supplement: Supplementary file 1 — Figure S1. Box‐plot highlighting the means of each parameter for the ‘Protection’ group between the understory (blue quantiles) and cave (green quantiles) environments. Mesophyll outer wall thickness (OWT); mesophyll epidermis quantity (ET); abaxial epidermis quantity (AEQ); adaxial epidermis quantity (ADQ). [file PLB-28-441-s002.jpg]

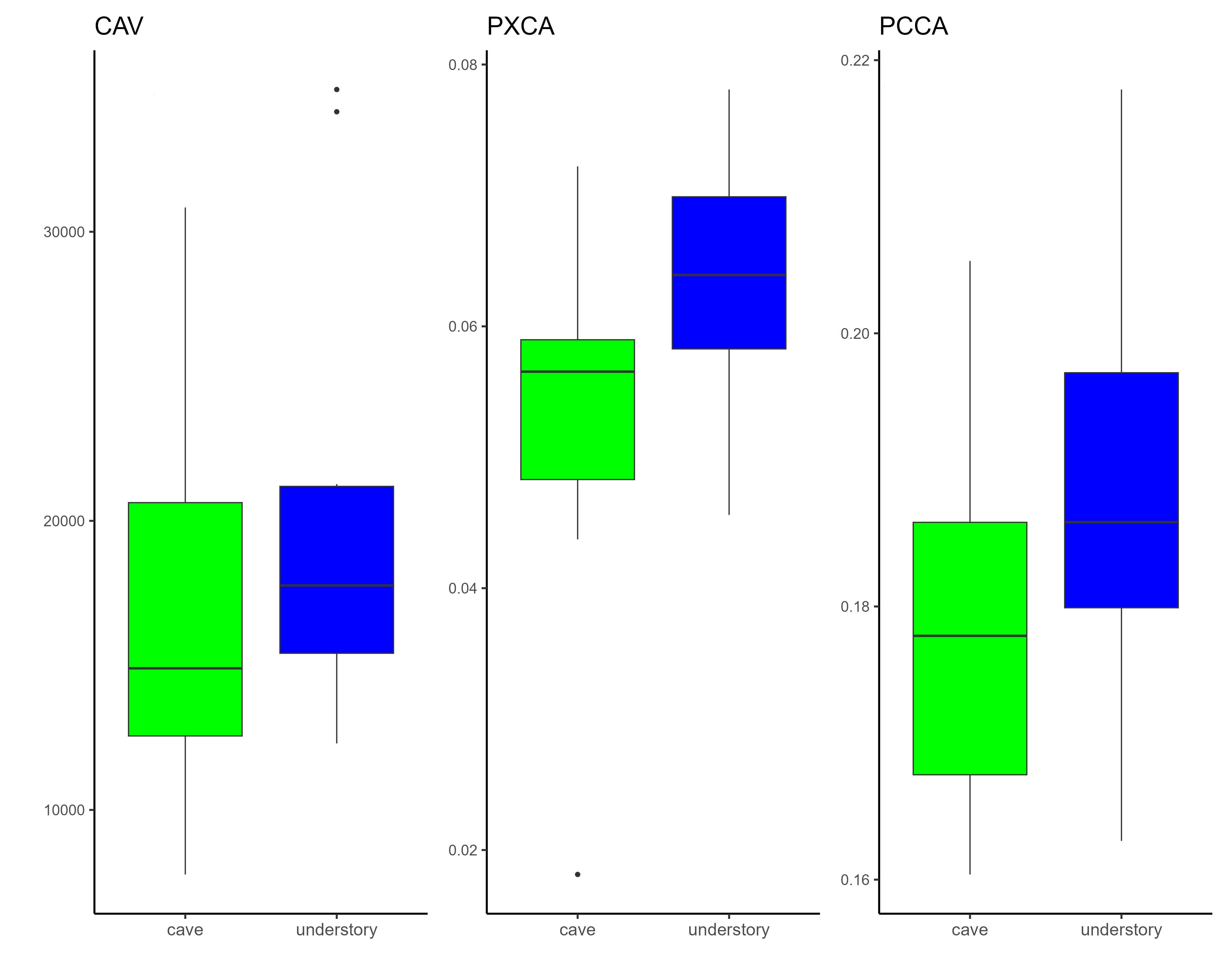

Supplement: Supplementary file 2 — Figure S2. Box‐plot highlighting the means of each parameter for the ‘Support’ group between the understory (blue quantiles) and cave (green quantiles) environments. Collenchyma area of the central vein (CAV); proportion of total xylem area to central vein area (PXCA); proportion of total collenchyma area to central vein area (PCCA). [file PLB-28-441-s001.jpg]
